# Relationship between the progression of posterosuperior rotator cuff tear size and shoulder abduction function: A cadaveric study *via* dynamic shoulder simulator

**DOI:** 10.3389/fbioe.2022.858488

**Published:** 2022-09-27

**Authors:** Liren Wang, Yuhao Kang, Haocheng Jin, Mingqi Wang, Yiyao Wei, Haihan Gao, Dingyi Shi, Suiran Yu, Guoming Xie, Jia Jiang, Jinzhong Zhao

**Affiliations:** ^1^ Department of Sports Medicine, Shanghai Jiao Tong University Affiliated Sixth People’s Hospital, Shanghai, China; ^2^ Regenerative Sports Medicine and Translational Youth Science and Technology Innovation Workroom, Shanghai Jiao Tong University School of Medicine, Shanghai, China; ^3^ School of Basic Medical Science, Fudan University, Shanghai, China; ^4^ Shanghai Jiao Tong University School of Medicine, Shanghai, China; ^5^ School of Mechanical Engineering, Shanghai Jiao Tong University, Shanghai, China; ^6^ Regenerative Sports Medicine Lab of the Institute of Microsurgery on Extremities, Shanghai Jiao Tong University Affiliated Sixth People’ Hospital, Shanghai, China

**Keywords:** shoulder stimulator, rotator cuff tear, abduction function, biomechanics, deltoid

## Abstract

Posterosuperior rotator cuff tear (PSRCT) is one of the most common shoulder disorders in elderly people’s daily life; however, the biomechanical relationship between PSRCT and shoulder abduction function is still controversial. In this study, a total of twelve freshly frozen cadaveric shoulders were included and tested in five conditions: intact rotator cuff, 1/3 PSRCT, 2/3 PSRCT, entire PSRCT, and global RCT. In each condition, extra load (0%, 45%, and 90% failure load) was sequentially added to the distal humerus, and the function of the remaining rotator cuff was mainly evaluated *via* the middle deltoid force (MDF) required for abduction. It is found that the peak MDF is required for abduction did not differ among the three PSRCT conditions (1/3 PSRCT: 29.30 ± 5.03 N, *p* = 0.96; 2/3 PSRCT: 29.13 ± 9.09 N, *p* = 0.98; entire PSRCT: 28.85 ± 7.12 N, *p* = 0.90) and the intact condition (29.18 ± 4.99 N). However, the peak MDF significantly differed between the global RCT (76.27 ± 4.94 N, *p* < 0.01) and all PSRCT and intact conditions. Under 45% failure load, the MDF of the entire PSRCT and global tear conditions were significantly increased compared with another status. With the 90% failure load, only the 1/3 PSRCT condition maintained the same shoulder function as the intact rotator cuff. These biomechanical testing jointly suggested that the weight-bearing ability of the shoulder significantly decreased as PSRCT progressed.

## Introduction

Posterosuperior rotator cuff tear (PSRCT) complaints are highly variable, with some patients exhibiting minimal symptoms and discomfort, while others exhibit pseudoparalysis or debilitating pain (Oh, et al., 2011; Rashid, et al., 2017). This controversy may result from the rotator cuff tear (RCT) size and the physical demand of individual patients (Keener, et al., 2017; Rizvi, et al., 2021). Mild PSRCT patients with minimal physical requirements might not notice any symptoms, while severe PSRCT patients with more physical demands suffer greatly in daily life (Kim, et al., 2019; Kwon, et al., 2019; Keener, et al., 2020). The former may respond favorably to non-operative treatment, and surgical intervention may be more suited for the latter. However, no biomechanical studies have tested these hypotheses. Therefore, it is unclear if a significant functional impediment would be observed in an originally compensable PSRCT shoulder under increased extra load; this information would be informative for determining suitable clinical treatment options.

To investigate the biomechanical relationship between PSRCT and shoulder function, a suitable biomechanical testing system is indispensable. The most commonly used biomechanical testing system is Instron. However, the machine only gets primary testing results, such as stiffness, number of cycles to failure, and maximum load rage at failure, which do not reflect the dynamic process of shoulder abduction (van der Meijden, et al., 2013). In addition, only uniaxial force could be applied to the muscle or tendon unit in this kind of machine, which is an oversimplification of the shoulder. Recently established biomechanical testing systems have made much improvement. In order to make the biomechanical testing platform closer to the clinical environment, some researchers have added a mechanical arm to the biomechanical testing system (Wellmann, et al., 2011). Through the mechanical arm, the shoulder can be put into a specific position to simulate the activities of the human shoulder joint. Moreover, the researcher can control the glenohumeral joint flexion and internal rotation activities by fixing the scapula and humerus, so as to detect the relatively complex biomechanical results including forward–backward translation and maximum internal rotation. Baumgartner et al. (2014) made a metal mechanical biomechanical testing system. They used an electric linear driver to simulate the force exerted by the deltoid, supraspinatus, infraspinatus/teres minor, and subscapularis, and transmitted it to the metal humerus through a cable pulley system, thus simulating the force exerted by the rotator cuff on the humerus. By equipping the metal machinery with sensors, the system can output accurate biomechanical results, including the force of each rotator cuff muscle and the glenohumeral contact force. Mihata et al. (2016) established a static biomechanical testing system, which would apply precise force on the deltoid, supraspinatus, infraspinatus, teres minor, and subscapularis to stimulate abduction motion when the glenohumeral joint angle was fixed at a certain angle (Mihata, et al., 2016). In this testing system, the shoulder index in each injured or repaired condition could be compared by evaluating the acromial contact area or glenohumeral contact force. Nevertheless, this is still a static biomechanical testing system, not reflecting the dynamic change of the shoulder. Thus, it is essential to establish a dynamic shoulder abduction stimulator that is able to reflect the active process of shoulder motion in order to precisely determine the biomechanical difference in each shoulder condition.

In this study, we explored the relationship between functional impediments of the shoulder and RCT size under extra load using a previously established dynamic shoulder abduction simulator. *Via* this machine, the tendinous insertions of the deltoid (anterior, middle, and posterior), infraspinatus/teres minor, supraspinatus, and subscapularis were dynamically loaded through a pneumatic actuator, which allows dynamic shoulder abduction from 0° to 90° (Video 1). We hypothesized that as the extra load increased, shoulder abduction impediments would be observed in a medium PSRCT, which was previously believed to be compensable.

## Materials and methods

### Specimen preparation and testing measurements

**FIGURE 1:**
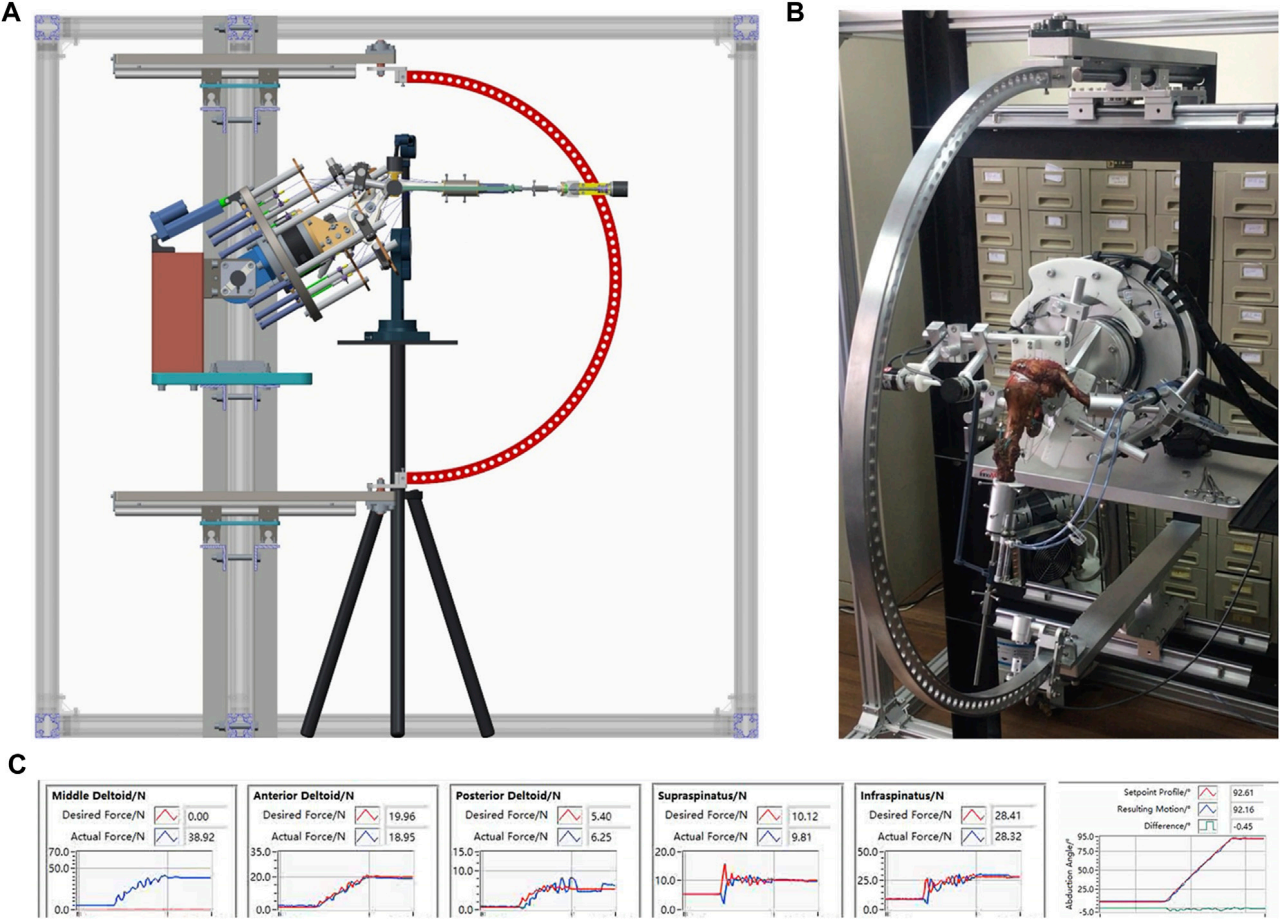
**(A)** Schematic and **(B)** actual experimental setup of the dynamic experimental shoulder biomechanics system, which allows 0°–60° glenohumeral abduction; **(C)** Dynamic mechanical detection of muscle force stimulation.

This study was reviewed by the Science and Research Development of the Shanghai Sixth People’s Hospital, which concluded that no institutional review was necessary for this research. Cadaver shoulders (donated for medical research from the tissue bank of our university; six males and six females; aged between 54–68 years) without the signs of abnormality or preexisting pathological findings, including a full-thickness RCT, osteoarthritis, or fatty infiltration, detected *via* computed tomography and gross visual examination, were used. The cadaver shoulders were thawed overnight before the experiment. Tendinous insertions of the rotator cuff and deltoid on the humerus were reserved. Other tissue was removed while the coracoacromial ligament and capsule were carefully retained. After preparation, each specimen was mounted on a previously validated biomechanical testing system (Figure 1) (Wang L. et al., 2021). To perform active dynamic evaluation, the tendinous insertion of the anterior deltoid, middle deltoid, posterior deltoid, superior and inferior subscapularis, superior and inferior supraspinatus, infraspinatus, as well as teres minor were attached to a specific actuator, prior to load application, as reported previously. Customized plates and nails were used to imitate physiological muscle force vectors without friction. Subsequently, we simulated active dynamic abduction from 0° to 90°, using scapular rotation, and adjusted to a 2:1 glenohumeral-to-scapulothoracic ratio.

### Testing model setup

Each specimen received dynamic glenohumeral abduction from 0° to 90° during our investigation. Shoulder muscle load distribution was assessed *via* load on the middle deltoid (supporting information). Peak middle deltoid force (MDF) referred to the peak value of MDF during shoulder dynamic abduction. Stable MDF referred to the value of MDF when the shoulder abduction angle is stabilized at 90°. Peak subacromial contact pressure (SACP) referred to the peak value of subacromial contact pressure during shoulder dynamic abduction recorded by a pressure measurement system (Fujifilm, Tokyo: measurement accuracy: 0.25 mPa). Average SACP referred to the average value of subacromial contact pressure during shoulder dynamic abduction recorded by pressure measurement system (Fujifilm, Tokyo: measurement accuracy: 0.25 mPa). Subacromial contact area (SACA) referred to the subacromial contact area during shoulder dynamic abduction recorded by a pressure measurement system (Fujifilm, Tokyo). Subacromial contact force (SACF) referred to the cumulative subacromial contact force during shoulder dynamic abduction recorded by a pressure measurement system (Fujifilm, Tokyo). Peak glenohumeral contact force (GHCF) referred to the peak value of GHCF during shoulder dynamic abduction. Stable GHCF referred to the value of GHCF when the shoulder abduction angle is stabilized at 90°. The value of GHCF was previously used to evaluate the shoulder stability on different rotator cuff injuries or repairing conditions *via* a static shoulder biomechanical testing system when the force applied to the shoulder is constant (Mihata, et al., 2012; Mihata, et al., 2016). However, in this dynamic biomechanical testing system, the force applied to the shoulder has significant differences in different rotator cuff injury conditions. To eliminate this difference, we used the GHCF/MDF ratio to represent the shoulder stability on different rotator cuff injuries or repairing conditions.

### Simulation of abduction failure

In biomechanical studies, it is indeed possible to make the shoulder complete 0–90° abduction with extra loading by increasing the force of the middle deltoid. However, if the increased force applied by the middle deltoid exceeds the threshold of normal deltoid in the human body, the 0–90° abduction can only be completed on the biomechanical machine, not in the human body. Therefore, the threshold of middle deltoid force is very important. In previous biomechanical studies, the failure load of the middle deltoid was usually set to 80 N. (Cline et al., 2021; Denard, et al., 2022; Tibone, et al., 2022). According to these suggestions, when installing the electric actuators matching the middle deltoid, we specially selected the electric actuators with a maximum range of 100 N. *Via* this electric actuator, when the biomechanical machine cannot complete 0–90° abduction, it means that the normal shoulder joint may not be able to complete the abduction of the shoulder joint in this certain situation, which is determined as a failure state in this study. In this condition, the maximal extra load was recorded as 100% failure load. In this study, 0, 45, and 90% failure loads were individually added to each shoulder on the distal humerus to stimulate daily activities as an empty hand, medium, and heavy upper extremity weight-bearing, respectively.

### Experimental conditions

**FIGURE 2:**
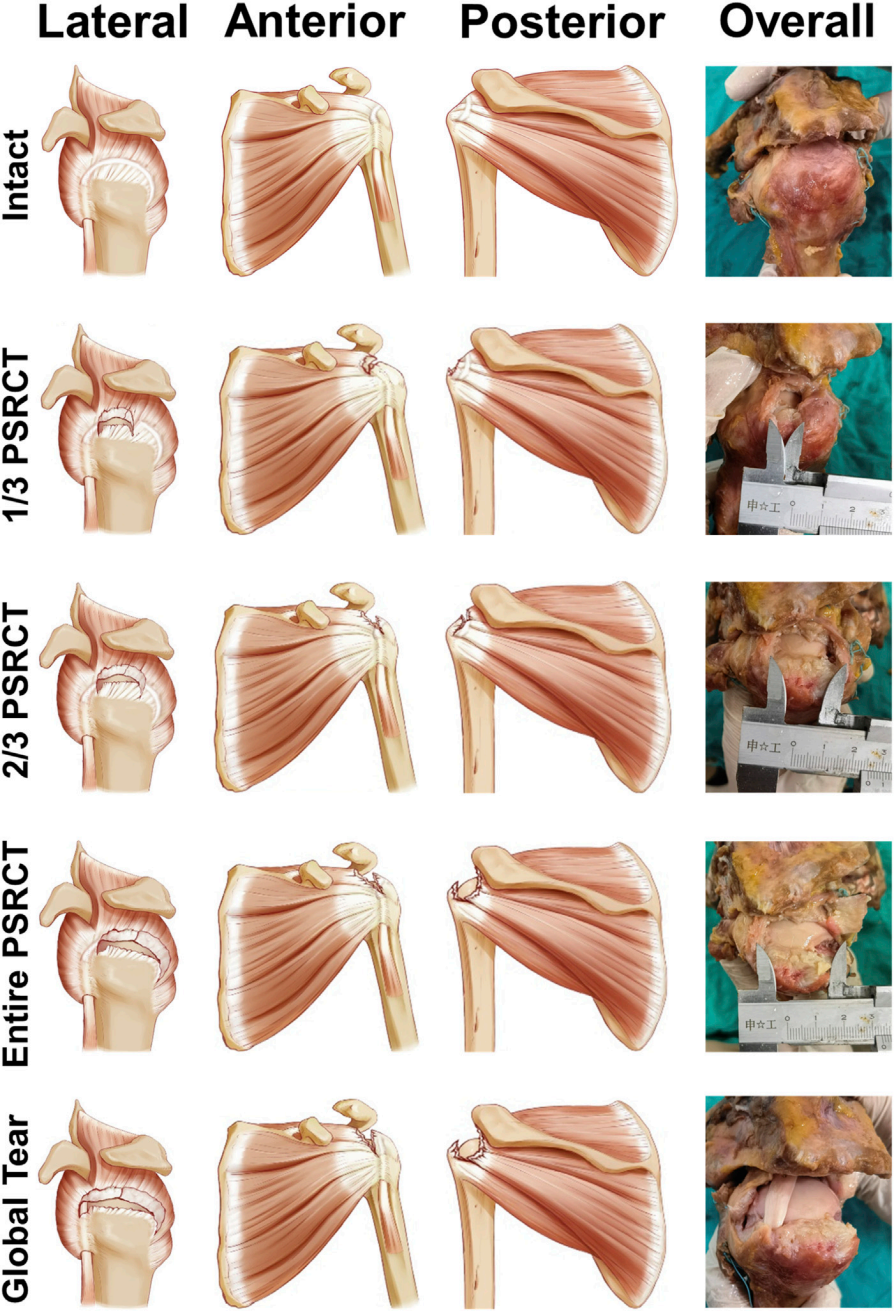
Schematic graph of the testing conditions.

In total, 15 conditions (five PSRCT conditions with three loads each) were tested (Figure 2). Intact rotator cuff shoulder was recorded as condition 1. Then, PSRCT was created from the anterior insertion of the supraspinatus with sequentially enlarged sizes to establish three PSRCT models: the anterior one-third (1/3 PSRCT, condition 2), anterior and middle one-third (2/3 PSRCT, condition 3), and entire posterior–superior rotator cuff (entire PSRCT, condition 4). The rotator cuff part of the teres minor and the subscapularis lying above the rotation center of the humeral head was torn as a global tear control (global tear, condition 5). For each condition, 0, 45, and 90% failure loads were individually added to the distal humerus to examine the function of shoulder abduction.

### Statistical analysis

The average of three measurements from each parameter was used for data analyses. To test differences in the peak MDF, stable MDF, average SACP, peak SACP, SACA, SACF, stable GHCF/MDF ratio, and peak GHCF/MDF ratio, two-way ANOVA analysis was performed. When a significant difference in interaction was observed, a simple effect analysis was performed. *p*-value of <0.05 was considered significant.

To reach 80% power based on the mean and standard deviation of the first four specimens, three specimens were required for stable MDF and the peak GHCF/MDF ratio, three specimens were required for SACA and the GHCF/MDF ratio, seven specimens were required for peak SACP, nine specimens were required for average SACP, ten specimens were required for peak MDF, and eleven specimens were required for SACF. Totally, twelve cadaveric specimens were used.

## Results

### Stimulation of pseudoparalysis in the biomechanical testing system

**FIGURE 3:**
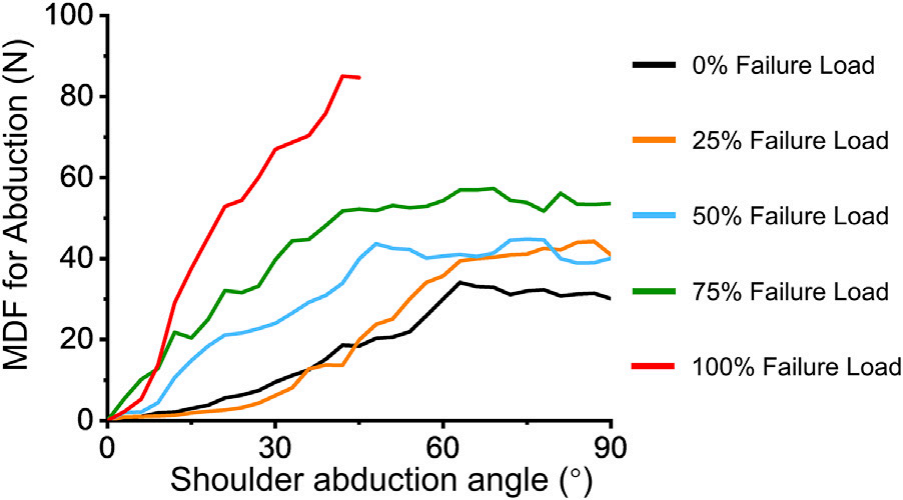
Effect of extra loading on MDF during abduction.

Clinically, pseudoparalysis patients suffer from a limited abduction angle. However, in a biomechanical testing system, with enough force and fulcrum, an arm can always abduct to a certain angle. Nevertheless, the deltoid is increasingly required to complete the abduction angle. Thus, instead of the maximum abduction degree, the MDF required for the abduction was considered the most important factor for evaluating pseudoparalysis in the current biomechanical testing system. Figure 3 presents the dynamic change between MDF abduction and the shoulder abduction angle. The minimum MDF required for abduction with 100% failure load less than 90° was 87.58 ± 7.17 N (95% confidence interval [CI]: 83.03–92.14 N), which was significantly increased compared with that for the 0% failure load condition (29.18 ± 4.99 N, 95% CI: 26.01–32.36 N).

### Effect of rotator cuff tear on middle deltoid force during abduction

**FIGURE 4:**
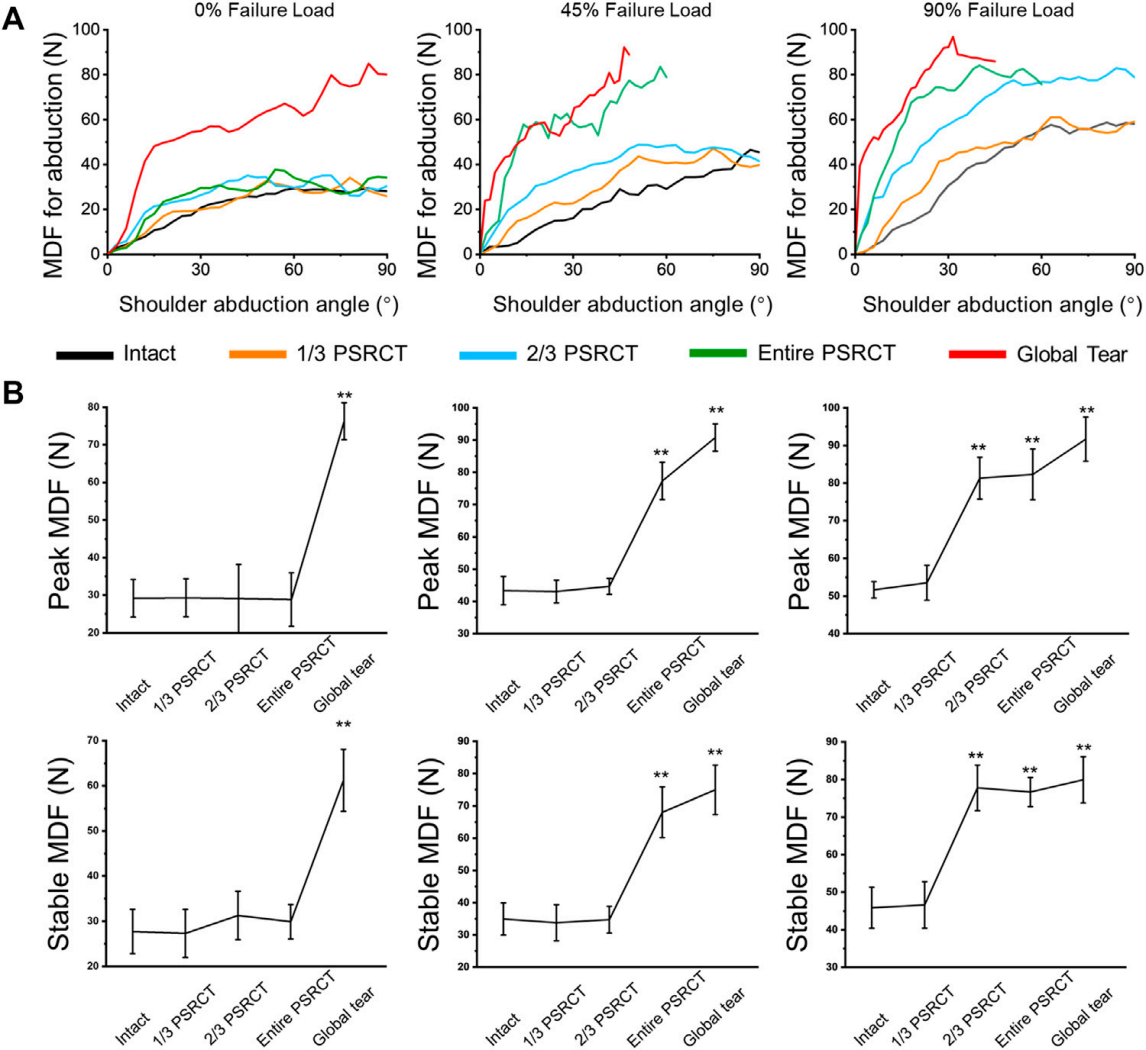
Effect of RCT on MDF during abduction. **(A)** The MDF curves of the intact, 1/3, 2/3, entire, and global tear conditions under 0, 45, and 90% failure loads. **(B)** The peak and stable MDFs during 60° glenohumeral abduction. MDF, middle deltoid force. **, a significant difference compared with the intact condition, *p* < 0.01.

Figure 4 presents the MDF curve of the intact and other RCT conditions under variable loading. For the 0% failure load condition (Table 1), the peak and stable MDFs in the global tear condition were significantly increased compared with those in the intact, 1/3 PSRCT, 2/3 PSRCT, and entire PSRCT conditions. For the 45% failure load condition (Table 1), the peak and stable MDFs in the entire PSRCT and global tear conditions were significantly increased compared with those in the intact, 1/3 PSRCT, and 2/3 PSRCT conditions. For the 90% failure load (Table 1), the peak and stable MDFs in the 2/3 PSRCT, entire PSRCT, and global tear conditions were significantly increased compared with those in the intact and 1/3 PSRCT conditions.

**TABLE 1:** Effect of RCT on MDF during abduction.

	0% Failure load (N)		45% Failure load (N)		90% Failure load (N)	
	Peak	Stable	Peak	Stable	Peak	Stable
Intact	29.18 ± 4.99	27.71 ± 4.93	43.35 ± 4.39^a^	34.91 ± 4.98^a^	51.66 ± 2.17^a,b^	45.84 ± 5.45^a,b^
1/3 PSRCT	29.30 ± 5.03	27.28 ± 5.33	43.09 ± 3.52^a^	33.77 ± 5.59^a^	53.52 ± 4.64^a,b^	46.62 ± 6.21^a,b^
2/3 PSRCT	29.13 ± 8.14	31.25 ± 5.38	44.68 ± 2.46^a^	37.20 ±4.02^a^	81.31 ± 5.57^a,b,^**	77.77 ± 6.04^a,b,^**
entire PSRCT	28.85 ± 7.12	29.88 ± 3.81	77.32 ± 5.81^a,^**	68.00 ± 7.84^a,^**	82.31 ± 6.74^a,^**	76.65 ± 3.86^a,b,^**
global tear	76.27 ± 4.94^**^	61.22 ± 6.88^**^	90.77 ± 4.23^a,^**	74.95 ± 7.65^a,^**	91.67 ± 5.85^a,^**	79.90 ± 6.14^a,^**

MDF: middle deltoid force; RCT: rotator cuff tear. ^a^significant difference compared with 0% failure load, *p* < 0.05; ^b^significant difference compared with 45% failure load, *p* < 0.05; **significant difference compared with the intact condition, *p* < 0.01.

### Effect of rotator cuff tear on the subacromial contact pressure, area, and force during abduction

**FIGURE 5:**
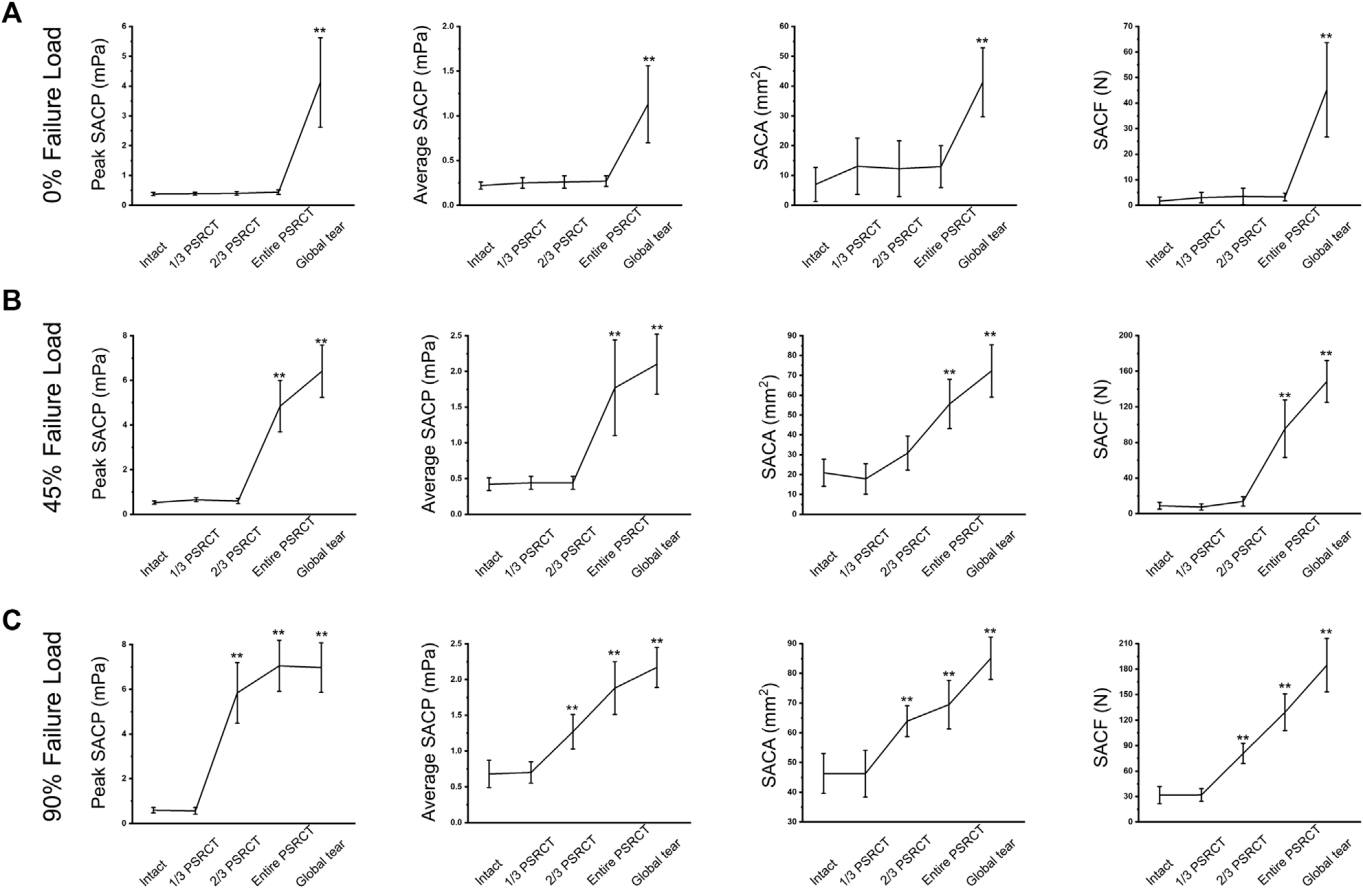
Effect of RCT on the peak and average SACPs, SACA, and SACF under **(A)** 0, **(B)** 45, and **(C)** 90% failure loads during abduction. SACP, subacromial contact pressure; SACA, subacromial contact area; SACF, subacromial contact force. **, a significant difference compared with the intact condition, *p* < 0.01.

As is shown in Figure 5, for the 0% failure load (Table 2), peak and average SACPs, SACA, and SACF in the global tear condition were significantly increased compared with those in the intact, 1/3 PSRCT, 2/3 PSRCT, and entire PSRCT condition. For the 45% failure load (Table 2), the peak and average SACPs, SACA, and SACF in the entire PSRCT and global tear condition were significantly increased compared with those in the intact, 1/3 PSRCT, and 2/3 PSRCT conditions. For the 90% failure load (Table 2), the peak and average SACPs, SACA, and SACF in the 2/3 PSRCT, entire PSRCT, and global tear conditions were significantly increased compared with those in the intact and 1/3 PSRCT conditions.

**TABLE 2:** Effect of RCT on the subacromial contact pressure, area, and force during abduction.

	0% Failure Load (N)				45% Failure Load (N)				90% Failure Load (N)			
	Peak SACP(MPa)	Average SACP(MPa)	SACA(mm^2^)	SACF(N)	Peak SACP(MPa)	Average SACP(MPa)	SACA(mm^2^)	SACF(N)	Peak SACP(MPa)	Average SACP(MPa)	SACA(mm^2^)	SACF(N)
Intact	0.38 ± 0.05	0.22 ± 0.04	7.00 ± 5.73	1.62 ± 1.60	0.52 ± 0.07 mPa^a^	0.42 ± 0.11^a^	20.92 ± 6.84^a^	8.91 ± 3.88^a^	0.59 ± 0.12^a,b^	0.68 ± 0.19^a,b^	46.33 ± 6.70^a,b^	31.59 ± 10.08^a,b^
1/3 PSRCT	0.40 ± 0.06	0.25 ± 0.06	13.08 ± 9.46	2.98 ± 2.05	0.65 ± 0.09^a^	0.44 ± 0.09^a^	17.83 ± 7.65	7.48 ± 3.41^a^	0.56 ± 0.15^a,b^	0.70 ± 0.15^a,b^	46.25 ± 7.90^a,b^	31.81 ± 7.56^a,b^
2/3 PSRCT	0.40 ± 0.06	0.26 ± 0.07	12.25 ± 9.37	3.43 ± 3.22	0.59 ± 0.12^a^	0.44 ± 0.09^a^	30.83 ± 8.59^a^	13.79 ± 5.28^a^	5.84 ± 1.36^a,b,^**	1.27 ± 0.24^a,b,^**	63.92 ± 5.20^a,b,^**	80.76 ± 11.83^a,b,^**
entire PSRCT	0.44 ± 0.08	0.27 ± 0.06	12.98 ± 7.03	3.22 ± 1.51	4.84 ± 1.15^a,^**	1.77 ± 0.67^a,^**	55.58 ± 12.41^a,^**	95.42 ± 32.33^a,^**	7.05 ± 1.14^a,b,^**	1.88 ± 0.37^a,^**	69.50 ± 8.17^a,b,^**	129.24 ± 21.52^a,b,^**
global tear	4.12 ± 1.50**	1.13 ± 0.43**	41.28 ± 11.56**	45.19 ± 18.49**	6.41 ± 1.17^a,^**	2.10 ± 0.43^a,^**	72.25 ± 13.14^a,^**	148.43 ± 23.54^a,^**	6.97 ± 1.11^a,^**	2.17 ± 0.28^a,^**	85.08 ± 7.15^a,b,^**	184.60 ± 31.44^a,b,^**

RCT: rotator cuff tear; SACA: subacromial contact area; SACF: subacromial contact force; SACP: subacromial contact pressure. ^a^significant difference compared with 0% Failure Load, *p* < 0.05; ^b^significant difference compared with 45% Failure Load, *p* < 0.05; **significant difference compared with the intact condition, *p* < 0.01.

### Effect of rotator cuff tear on the glenohumeral contact force/middle deltoid force ratio

**FIGURE 6:**
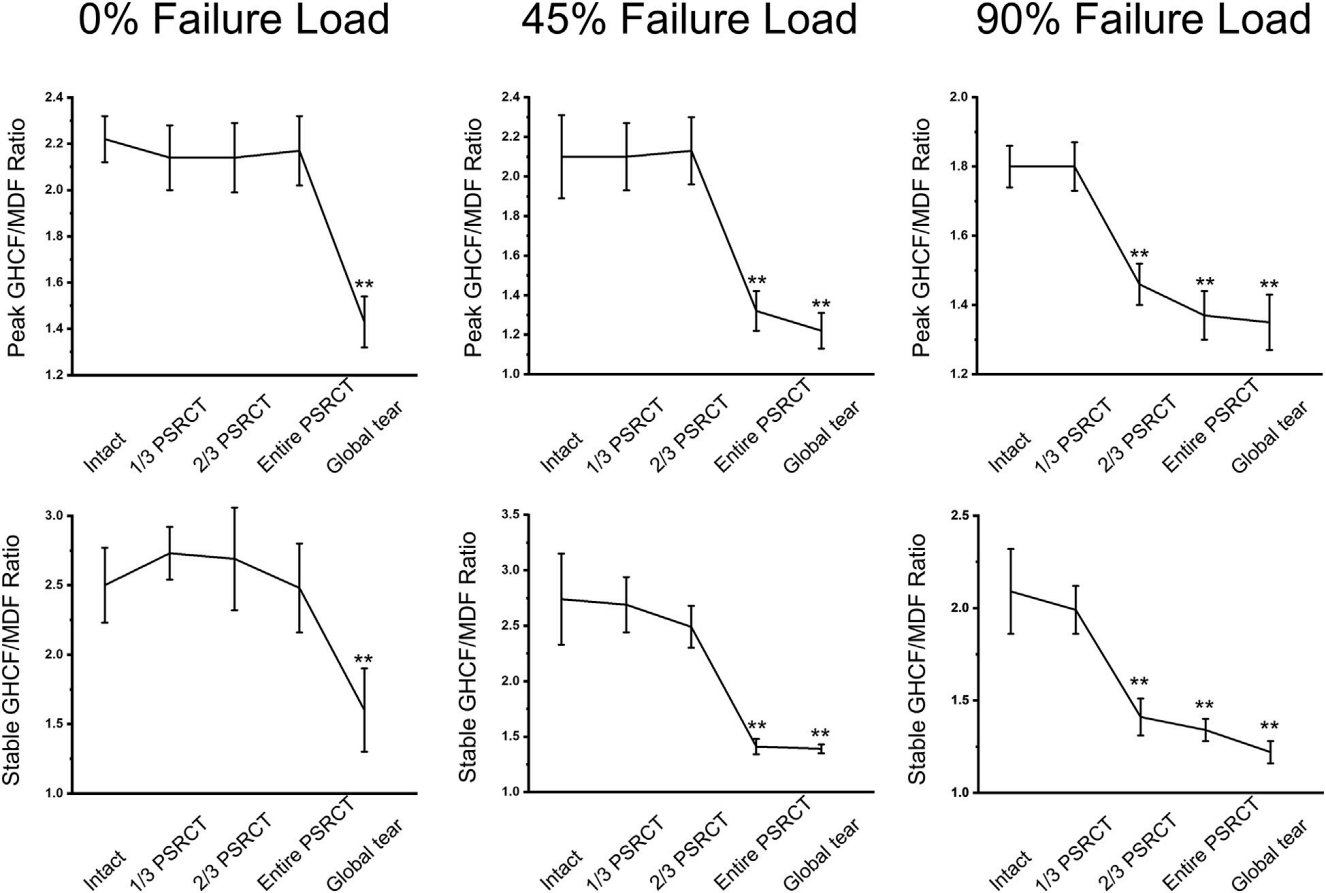
Effect of RCT on the peak and stable GHCF/MDF ratios under 0, 45, and 90% failure loads during abduction. MDF, middle deltoid force. **, a significant difference compared with the intact condition, *p* < 0.01.

The GHCF/MDF ratio is used to evaluate shoulder stability based on a study (Figure 6) (Wang L. R. et al., 2021). For the 0% failure load (Table 3), the peak and stable GHCF/MDF ratios in the global tear condition were significantly decreased compared with those in the intact, 1/3 PSRCT, 2/3 PSRCT, and entire PSRCT conditions. For the 45% failure load, the peak and stable GHCF/MDF ratios in the entire PSRCT and global tear conditions were significantly decreased compared with those in the intact, 1/3 PSRCT, and 2/3 PSRCT conditions. For the 90% failure load, the peak and stable GHCF/MDF ratios in the 2/3 PSRCT, entire PSRCT, and global tear conditions were significantly decreased compared with those in the intact and 1/3 PSRCT conditions.

**TABLE 3:** Effect of RCT on the GHCF/MDF ratio.

	0% Failure Load (N)		45% Failure Load (N)		90% Failure Load (N)	
	Peak	Stable	Peak	Stable	Peak	Stable
Intact	2.19 ± 0.12	2.55 ± 0.29	2.11 ± 0.22	2.74 ± 0.41	1.80 ± 0.06^a,b^	2.09 ± 0.23^a,b^
1/3 PSRCT	2.14 ± 0.14	2.73 ± 0.19	2.11 ± 0.17	2.69 ± 0.25	1.80 ± 0.07^a,b^	1.99 ± 0.13^a,b^
2/3 PSRCT	2.14 ± 0.15	2.69 ± 0.38	2.13 ± 0.17	2.49 ± 0.19	1.46 ± 0.06^a,^**	1.41 ± 0.10^a,^**
entire PSRCT	2.17 ± 0.15	2.48 ± 0.32	1.32 ± 0.15^a,^**	1.41 ± 0.07^a,^**	1.37 ± 0.07^a,^**	1.34 ± 0.06^a,^**
global tear	1.43 ± 0.11**	1.60 ± 0.30**	1.22 ± 0.09^a,^**	1.39 ± 0.04^a,^**	1.35 ± 0.08^a,^**	1.22 ± 0.06^a,b,^**

GHCF: glenohumeral contact force; MDF: middle deltoid force; RCT: rotator cuff tear. ^a^significant difference compared with 0% Failure Load, *p* < 0.05; ^b^significant difference compared with 45% Failure Load, *p* < 0.05; **significant difference compared with the intact condition, *p* < 0.01.

## Discussion

Our biomechanical findings indicated that as PSRCT progressed, the weight-bearing ability of the shoulder became significantly impaired. There was a significant increase in the peak and stable MDFs, peak and average SACPs, SACA, and SACF with extra loading compared with the empty hand condition. Furthermore, there was a significant decrease in shoulder stability in high–weight-bearing conditions with larger tears, indicated by decreased peak and stable GHCF/MDF ratios. Taken together, these results suggested that when there is some remaining rotator cuff attachment above the equatorial line of the humeral head, active abduction is not affected by the PSRCT without extra loading, regardless of the tear size. However, with the extra load, the MDF significantly increases, resulting in the dysfunction of the remnant rotator cuff attachment above the equatorial line of the humeral head. Thus, the humeral head inevitably shifts upwards, significantly impairing the glenohumeral abduction function.

Recent studies have investigated whether a critical RCT stage contributed to the functional impediments of shoulder abduction function. Oh et al. (2011) suggested that the entire detachment of supraspinatus was the critical RCT stage causing significantly decreased abduction capability and increased anterior-posterior humeral head shift. The impeded shoulder function deteriorated when the infraspinatus was subsequently detached. However, Dyrna F et al. (2018) investigated biomechanical differences in deltoid force after posterior-superior and anterior-superior massive RCTs (MRCTs) in a cadaveric model using a sub dynamic testing system and found that the mean force generated by anterior, middle, and posterior deltoids significantly increased in anterior-superior MRCT but not in posterior-superior MRCT. Yoon TH et al. (2019) enrolled 108 MRCT patients and found that the patients with subscapularis and teres minor integrity experienced significantly decreased incidences of conventional treatment failure compared with patients lacking integrity of one or both muscles. Currently, the relationship between the abduction limitation and global RCT is under review. Ernstbrunner et al. (2021) reviewed 50 RCT patients and found that without extra loading, the shoulder abduction function only deteriorated when the degree of global tear extension reached 225 ± 14°. These contract biomechanical findings might be because PSRCT might partly impair the rotator cuff function compared with native uninjured shoulder, which can be detected in intricate biomechanical testing. However, the whole glenohumeral dynamic abduction remained unchanged without bearing extra loading.

**FIGURE 7:**
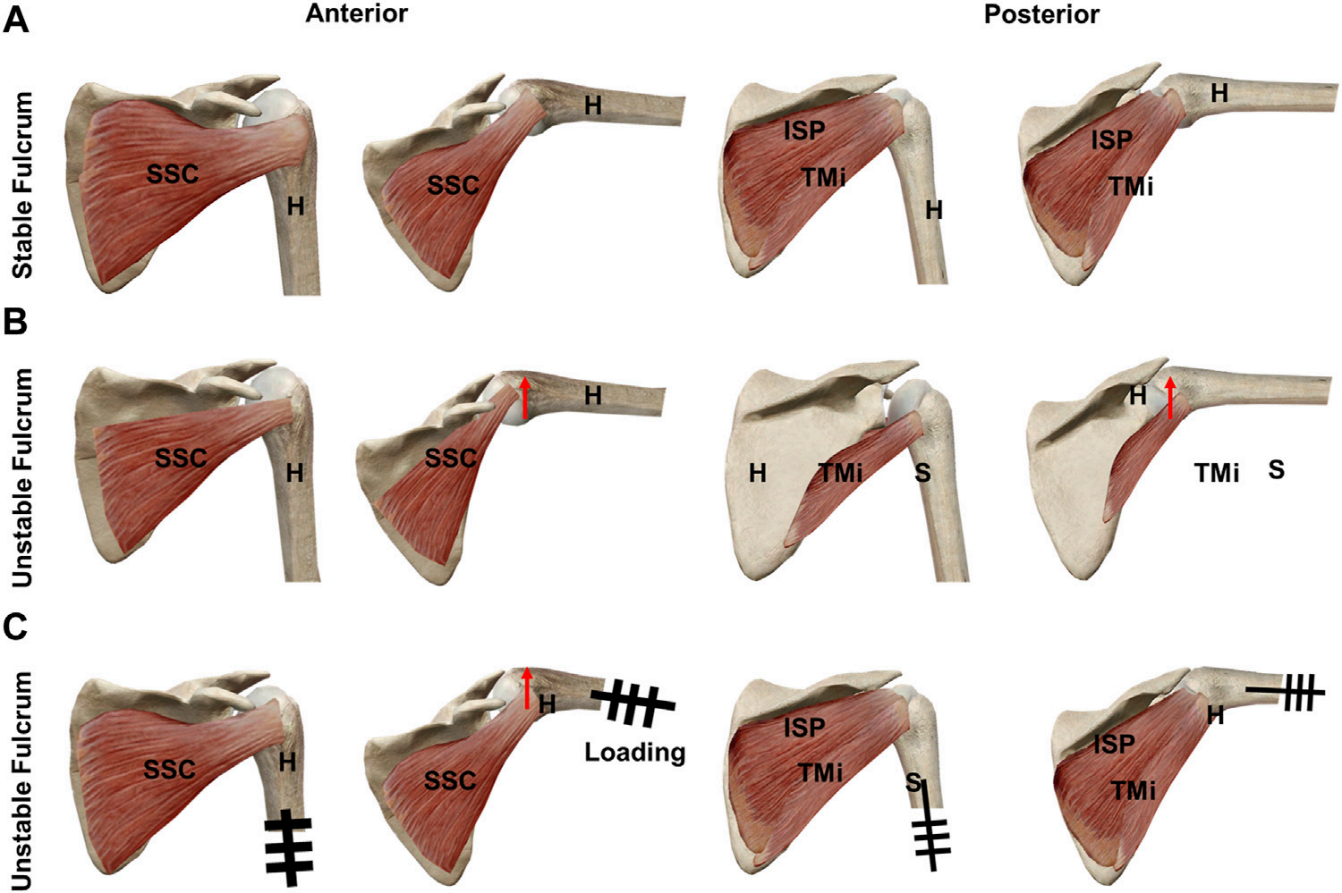
Schematic graphs of the superior migration of the humeral head and fulcrum during abduction: **(A)** RCT with remnant rotator cuff tissue above the equatorial line of the humeral head at 0° and 60° glenohumeral abduction, **(B)** 0° and 60° glenohumeral abduction of MRCT without remnant rotator cuff tissue above the equatorial line of the humeral head, and **(C)** RCT with remnant rotator cuff tissue above the equatorial line of the humeral head with extra loading at 0° and 60° glenohumeral abduction. H: humerus; ISP: infraspinatus; RCT: rotator cuff tear; S: scapular; SSC: subscapularis; TMi: teres minor.

Clinically, MRCT patients always complain about heavy lifting limitations in daily life. However, the abduction strength is also important in the Constant–Murley Score (Hirschmann, et al., 2010; Roy, et al., 2010). Our biomechanical results suggested that the loss of muscle tone resulted in a limited abduction ability for carrying heavy things and increased the required MDF for abduction. This biomechanical phenomenon might be explained by the fulcrum theory. Burkhart et al. (1992) suggested that the glenohumeral fulcrum is classified into stable and unstable fulcrums depending on the severity of RCT. The unstable fulcrum was commonly caused by MRCT and clinically characterized as the decreased interface between the humeral head position and acromion on magnetic resonance images or radiographs (de Oliveira Franca et al., 2016; Denard, et al., 2018) which was believed to play a critical role in glenohumeral function (Kozono, et al., 2018a; Kozono, et al., 2018b). The shift from a stable to an unstable fulcrum represented a significant alternation of the normal biomechanical status and was correlated with impaired abduction function. However, because the decreased interface between the humeral head position and the acromion is not easy to record in dynamic biomechanical testing, the SACP, SACA, and SACF were used to represent the fulcrum status. The increased SACP, SACA, and SACF indicated increased contact between the humerus and acromion, suggesting a shift to an unstable fulcrum. The present study demonstrated that the glenohumeral fulcrum remained stable in the 1/3 PSRCT, 2/3 PSRCT, and entire PSRCT conditions. However, as the extra load added on the distal humerus increased, the remnant rotator cuff above the equatorial line of the humeral head gradually became inadequate for stabilizing the humeral head. Consequently, the humeral head migrated proximally and hit the acromion, increasing SACP, SACA, and SACF and forming a newly unstable fulcrum (Figure 7). We hypothesized that unlike the stable fulcrum, the newly formed unstable fulcrum would result in significantly increased MDF during the abduction, as previously suggested.

The present study indicated a negative relationship between the PSRCT and shoulder function without extra loading and a positive correlation between the tear size and shoulder abduction limitations as the extra load increased. Thus, RCT treatment should be matched with the physical demand of the patient (Kweon, et al., 2015; Kim, et al., 2018; Ramme, et al., 2019; Merlet, et al., 2021). For most young patients with sports needs, a surgical repair to restore the rotator cuff integrity is inevitable to guarantee the quality of life after injury (Klouche, et al., 2016; Azzam, et al., 2018; Rossi, et al., 2019). However, for some elderly patients with medium or entire PSRCT who only require daily life movements, such as hair brushing, conservative treatment with an analgesic might be adequate.

There were some limitations to our study. First, only deltoid, supraspinatus, infraspinatus, subscapularis, and teres minor were loaded during our humeral abduction experiments. Other muscles, including the pectoralis major, latissimus dorsi, and teres major, should be included in future dynamic biomechanical studies. Second, this biomechanical testing system was based on a pneumatic loading machine. Third, in this study, the relative force ratio of each group of muscles was consistent during the 0–90° shoulder abduction, in the state of rotator cuff intact, injury, and repairing, which may not be identical to the clinical situation. Forth, resulting from the limitation of feedback speed of the biomechanical testing system, the profile of the MDF during 0–90° shoulder abduction is not smooth enough, which might affect the reliability of the conclusion. Fifth, the dynamic muscle loading protocol in this biomechanical testing system was based on normal shoulder conditions, which are different compared with RCT conditions and might influence the biomechanical results.

## Data Availability

The raw data supporting the conclusion of this article will be made available by the authors, without undue reservation.
